# Complementary and alternative medicine use in coronary heart disease patients: a cross-sectional study from Palestine

**DOI:** 10.1186/s12906-020-03028-w

**Published:** 2020-07-20

**Authors:** Abdelraouf O. Salah, Ahmad D. Salameh, Mohanad A. Bitar, Sa’ed H. Zyoud, Abdulsalam S. Alkaiyat, Samah W. Al-Jabi

**Affiliations:** 1 Department of Medicine, College of Medicine and Health Sciences, An-Najah National University, Nablus, 44839 Palestine; 2 Poison Control and Drug Information Center (PCDIC), College of Medicine and Health Sciences, An-Najah National University, Nablus, 44839 Palestine; 3 Department of Clinical and Community Pharmacy, College of Medicine and Health Sciences, An-Najah National University, Nablus, 44839 Palestine; 4 Clinical Research Centre, An-Najah National University Hospital, Nablus, 44839 Palestine; 5 Public Health Department, College of Medicine and Health Sciences, An-Najah National University Hospital, An-Najah National University, Nablus, 44839 Palestine

**Keywords:** Complementary and alternative medicine, Coronary heart disease, Attitudes, Palestine

## Abstract

**Background:**

There is a lack of data on the use of complementary and alternative medicine (CAM) in patients with coronary heart disease (CHD). This study examined the use of CAM among patients with CHD, the reasons and factors influencing their use, the types of CAM used, and the relationship between patient’s demographics and the use of CAM.

**Methods:**

In order to determine the prevalence and usage of CAM among Palestinian patients with CHD, a cross-sectional descriptive study was performed from three different hospitals. Using a convenient sampling method, a questionnaire was completed in a face-to-face interview with the patients. Descriptive statistics were used for socio-demographic, and clinical variables. Siahpush scale was used to examine the attitude of CHD patients toward CAM use.

**Results:**

Of the 150 patients that were interviewed, 128 (85.3%) of the patients completed the questionnaire. The majority of CAM users reported CAM use for health problems other than CHD, while a total of 59 (45.9%) patients have used CAM for their heart problems. On the other hand, it was found that the place of residency and pattern of CHD were significantly associated with CAM use (*p* = 0.039 and 0.044, respectively). In addition, religious practices were found to be the most common form of CAM used by patients, while body and traditional alternative methods were the least being used. A significant association between the attitudes of patients with CHD and their use of CAM was found (patients’ attitudes towards alternative medicine and natural remedies were *p* = 0.011 and 0.044, respectively).

**Conclusions:**

CAM use among our respondents is common. Despite a lack of evidence-based research supporting its potential benefits and side effects. Understanding the factors that affect CAM use by CHD patients offers healthcare workers and policymakers an opportunity to better understand CAM use and ultimately improve patient-physician interactions.

## Background

Cardiovascular disease (CVD) is known to be the number one cause of death in both men and women in the United States [1, 2]. It has an annual mortality rate of approximately 600,000 people, which represents one in every four deaths [1]. Coronary heart disease (CHD) is the most prevalent form of CVD and accounts for more than 358,000 deaths yearly, costing the United States $108.9 billion each year. The most common risk factors for CVD include hypertension, elevated low density lipoprotein (LDL)-cholesterol, cigarettes, diabetes, obesity, physical inactivity, poor diet, and heavy use of alcohol [3–5]. CHD is the world’s leading cause of death, accounting for one-third of deaths for people worldwide [6]. CHD, an interchangeable concept with coronary artery disease, is a common term for plaque build-up in the coronary arteries, limiting blood flow to cardiac muscles and leading to ischemia [7].

According to the Palestinian Ministry of Health in 2010, heart disease is still considered the leading cause of death in Palestine, accounting for a one-quarter of all deaths, with cerebrovascular disease, cancer, and diabetes in the second, the third, and the fourth ranks, respectively [8]. In 2014 alone, CHD was responsible for 2.3% of deaths. It’s worth mentioning that CHD is the most prevalent form of CVD, accounting for about 36.1% of all cardiovascular-related deaths in the West Bank [9].

The use of complementary and alternative medicine (CAM) was found to be more prevalent among the younger population, females, Asians, and those with higher socioeconomic status [10–12]. In addition, the role of ethnicity has been recognized as the most useful predictor of CAM usage according to a study that was carried out in Trinidad and Tobago (SWRHA), along with the roles of friends, family, and religion [13]. The definition of CAM still is a controversial concept due to the wide variety of methods that can be included in its definition [14], which can be considered as one of the reasons why CAM usage prevalence is underestimated. For this reason, in the current study, the popular CAM methods used in Palestine were integrated into the list of options that were carefully explained to the respondents in face-to-face interviews in order to try and estimate, as accurately as possible, the prevalence of CAM use. In a previous study done in Trinidad, which was the first one to shed light on this subject, the definition of CAM was taken from the National Center for Complementary and Integrative Health as “a group of diverse medical and healthcare systems, practices, and products that are not generally considered part of conventional medicine” [13].

CAM, in particular herbal medicine, is proved to be effective in managing cardiovascular problems [15]. However, there is much evidence showing some interactions between conventional CHD medications and some herbal medicines [16–19]. Previous studies on the use of CAM by patients with CHD have been implemented in the healthcare field outside Palestine. The majority of studies were from the United States [20–29]. Results from the 2002 National Health Interview Survey [29], in which 10,572 patients with CVD were interviewed, revealed that 36% of patients had used CAM (excluding prayer) in the previous 12 months. Herbal compounds (18%) and mind-body treatments (17%) were the most widely used therapies. Other works have been carried out in the UK [14], China [30], Canada [31–33], Spain [34], Norway [35], Malaysia [36], and Germany [37] and results have shown a wide range of CAM usage (from 12 to 85%).

In spite of the pervasive use of CAM worldwide, in different illnesses and populations; there is a lack of studies that focus on its usage among Palestinians, which may have a significant effect on self-management among patients with CHD [38]. To our knowledge, multiple studies were carried out considering CAM use in situations and diseases other than CHD, including hypertension [39], diabetes mellitus [40], haemodialysis [41], cancer [42], or even the general public [38]. In addition, several studies have been carried out regarding herbal therapies among different populations, such as cancer patients [43, 44], university students [45], geriatric patients [46], and pregnant women [47, 48]. Despite many types of CAM, such as religious practices and traditional herbs, being an integral part of the culture, similar studies elaborating CAM usage by patients with CHD in Palestine could not be found [49–54].

Many patients use CAM from a traditional or cultural point of view, so we aimed to study this phenomenon from a scientific background. Therefore, the current study aimed to 1) determine the prevalence of CAM usage among patients with CHD and the physician’s role in introducing them to the patients in Palestine; 2) identify the most frequent categories of CAM used among Palestinian CHD patients; 3) investigate patients’ attitudes and beliefs towards CAM; 4) determine factors associated with CAM usage from different socio-demographic variables; 5) identify the main sources of information recommending the use of CAM; and 6) explore the patient-physician relationship regarding the usage of CAM.

Patients with CHD have to learn how to improve their lifestyle and to obtain their informal methods of self-management, which they believe can maximize their quality of life and prevent recurrence and deterioration of the disease, despite the absence of supporting evidence indicating its effectiveness. Given the global initiative on evidence-based medicine and the lack of raw data on how to use CAM safely and effectively, healthcare professionals and policymakers have become more cautious about the use of CAM among patients with CHD. Understanding patterns of CAM therapies in this study helps to make an original contribution in many areas including: 1) the clinical setting by providing more informed clinical care; 2) assisting policymakers in drafting the appropriate template for future policies; and 3) encouraging researchers to conduct more research on this topic in order to discover more clinically proven risks and benefits of CAM usage.

## Methods

### Study design

An analytical cross-sectional study was used to evaluate the prevalence of CAM usage among Palestinian patients with CHD and their attitudes towards it.

### Study setting and population

The study was carried out in the mid-northern region of the West Bank (Ramallah, Nablus) in the following hospitals: An-Najah University National Hospital, Al-Watani Hospital, and Ramallah Medical Complex.

### Sample size and sampling technique

According to the Ministry of Health reports of 2017 in Palestine [55], the average number of patients with CHD who attend the hospitals (study area population) is 240. This number was used as a guide for determining the sample size required for this analysis. By assuming a 50% expected response distribution for CAM use and allowing a 5% error margin at 95% confidence interval, the appropriate sample size for the analysis was calculated using a Raosoft sample size calculator: http://www.raosoft.com/samplesize.html. A total of 150 patients were approached according to sample size calculation; however, we got consent from 128 patients with a valid response of 85%. A convenience sampling technique was used to select participants from October 2018 to February 2019.

### Inclusion and exclusion criteria

Patients who were chosen to participate in our study had met the following inclusion criteria: 1) age ≥ 18 years; 2) a history of ischemic heart disease or coronary revascularization; and 3) no history of myocardial infarction in the previous 6 months. On the other hand, patients who had a debilitating condition that made them unfit to participate in the interviewing process were excluded.

### Data collection instrument

Face-to-face interviews were done using a questionnaire in native Arabic language based on similar previous studies [14, 41, 56–59].

The data collection instruments contained five sections (Additional file 1):

- The first section focused on the socio-demographic data provided by participants, such as age, gender, residence, marital status, level of education, occupation, income, height and weight. The body mass index (BMI) of each participant was calculated using the Excel program using the height and weight information provided by the participant.
- The second section recorded the duration of illness in years, the number of associated chronic morbid conditions, and smoking.
- The third section mainly focused on the consumption, frequency and methods of CAM usage. CAM treatments have been classified as follows: (1) traditional medical therapies, such as acupuncture; (2) biologically derived therapies, such as folk medicine, vitamins or other forms of herbal products; (3) manipulative and body-based approaches, such as massage or physical therapy; and (4) mind-body treatments, such as meditation. Exorcism (ruqya) was included as a mind-body treatment, in a fashion similar to previous studies [41, 56, 57, 60].

This study was designed to include all types of CAM methods used for the sole purpose of managing health conditions during the period of treatment of the disease. The frequency of CAM usage was reported per week. Additionally, our study focused on herbal medicine due to a high incidence of reported usage among the different populations [13, 61–63].

- In the fourth section, respondents were asked to provide reasons for using CAM in self-therapy practices using closed-ended questions.
- The last (fifth) section of the questionnaire was designed to assess patient’s attitudes towards the use of CAM based on the Siahpush score [58, 64], which included eight categories: attitudes toward alternative medicine (max score = 25), dissatisfaction with medical outcomes (max score = 30), dissatisfaction with medical encounters (max score = 35), individual responsibility (max score = 15), natural remedies (max score = 30), holism (max score = 20), rejection of authority (max score = 20) and consumerism (max score = 5). Questions were scored on a 5-point Likert scale (strongly agree, agree, don’t know, disagree, and strongly disagree) where “strongly agree” was given 5 points, and “strongly disagree” was given 1 point.

### Validity and reliability study

A panel of three professional pharmacists who are experts in CAM research examined and evaluated the face and content of the final questionnaire and evaluated the organisation, word meaning, medical terms, appropriateness, completeness and a consistent sequence of statements. After the questionnaire was finalized, the reliability and clarity of the questions among 10 CHD patients were pre-tested. During our pilot study, we managed to inspect the popular CAM methods that were used in Palestine, which helped us improve our orientation regarding which methods we should focus on and include in our study. Cronbach’s alpha was used to determine internal consistency. Cronbach’s alpha coefficients were acceptable for attitudes towards alternative medicine (α = 0.696), dissatisfaction with medical outcomes (α = 0.632), dissatisfaction with medical encounters (α = 0.626), individual responsibility (α = 0.904), natural remedies (α = 0.716), holism (α = 0.647), and rejection of authority (α = 0.610).

### Ethical considerations

Before this study was launched, all elements of the research procedure were approved by the Institutional Review Board (IRB) and local health administrators. Written consents were obtained from patients before the interviews. We explained that the collected information would only be used for research purposes, and its confidentiality would be preserved.

### Statistical analysis

Data were entered and analysed using the Statistical Package for Social Sciences (SPSS) version 18.0. Descriptive statistics were used for socio-demographic variables, medical history variables and CAM usage: frequencies and percentages of categorical variables, and mean and standard deviation for numerical variables. BMI was computed by dividing weight (kg) by (height (m))^2^ then the variable was categorized as: underweight if BMI < 18.50, normal (18.50–24.99), overweight (≥25), and obese (≥30). Factors associated with CAM usage were analyzed using the Chi-square test.

Descriptive analysis was performed to find CHD patients’ attitudes towards CAM. Attitudes to CAM and health were estimated using the Siahpush questionnaire; each question was scored on a five-point Likert scale, (strongly agree, agree, don’t know, disagree, strongly disagree) where strongly agree was given 5 points, and strongly disagree was given 1 point. The normality of the data was tested using the Kolmogorov-Smirnov test, and data were presented as median (interquartile range, IQR) for not normally distributed data. Factors associated with patients’ attitudes towards CAM were tested using the Man-Whitney test or Kruskal-Wallis test as appropriate. A value of *p* < 0.05 was considered statistically significant.

## Results

### Socio-demographic characteristics of the study participants

A total of 128 patients completed the questionnaire. The mean age of participants was 60.58 ± 10.86 years, with around two-thirds (67.2%; *n* = 86) being male. Almost half of the respondents were aged 51–65 years (*n* = 64; 49.6%), and (*n* = 43, 33.6%) were older than 65 years. The socio-demographic characteristics of the participants are given in Table 1. Regarding the place of residency, 68 (53.6%) lived in villages, 44 (34.7%) lived in cities and the rest lived in refugee camps. Almost half of the respondents were not working (*n* = 61, 48%), a quarter of them reported being retired (*n* = 31, 24%), 19 (14.4%) were private-sector employees and the rest were public sector employees (*n* = 17, 14.4%). When reporting obesity based on BMI calculations from self-reports, 33% (*n* = 42) were found to be obese, 42% (*n* = 54) were found to be overweight, 24.1% (*n* = 31) were found to be normal and only 1 patient was found to be underweight.

**Table 1:** Frequencies of categorical socio-demographic variables

Variable	Categories	Numbers^a^	%
**Gender**	Male	86	67.2
	Female	42	32.8
**Birthplace**	Palestine	122	95
	Outside	6	5
**Place of residency**	Village	68	53.6
	City	44	34.7
	Refugee camp	16	11.9
**Age intervals**	< 35	2	1.8
	36–50	19	15
	51–65	64	49.6
	> 65	43	33.6
**Work status**	Not working	61	48
	Government employee	17	13.6
	Private sector employee	19	14.4
	Retired	31	24
**Monthly income**	Less than 2000 NIS	67	52.2
	2000–5000 NIS	49	38.1
	5000–10,000 NIS	8	6.2
	More than 10,000 NIS	4	3.5
**Educational level**	High school	85	66.7
	Diploma	19	15
	Bachelor’s degree	23	17.5
	Higher studies	1	.8
**Smoking status**	Smoker	55	42.9
	Non-smoker	73	57.1
**Obesity**	Underweight	1	.9
	Normal	31	24.1
	Overweight	54	42.0
	Obese	42	33.0

NIS: new Israeli shekel (1 New Israeli Shekel = 0.29 US Dollar)

^a^There were missing data on some of the variables

### Medical history characteristics of the study participants

It was found that 93 (72.8%) of respondents were rehabilitated in the hospital. As for the pattern/type of CHD, it was reported to be Percutaneous Transluminal Coronary Angioplasty (PTCA) for 88 (68.7%) of the respondents, Coronary Artery Bypass Graft (CABG) for 20 (15.65%), and unstable angina for 20 (15.65%). Duration of illness was estimated to be less than 5 years for 74 (57.7%) of the respondents, 5–10 years for 37 (28.5%) of the respondents, 11–20 years for 14 (11.4%) of the respondents, and more than 20 years for 3 (2.4%) of the respondents. Regarding the associated disorders, around one-third (*n* = 42, 33%) of patients had hypertension, 34 (26.4%) had diabetes mellitus, 28 (21.7%) had peripheral vascular disease, and the rest (18.9%) had other disorders, such as respiratory problems, renal problems, and others. Furthermore, 101 (78.9%) of the respondents reported a history of emergency admission due to their underlying heart disease. Table 2 shows the frequencies and percentages regarding the medical history of the respondents.

**Table 2:** Frequencies and percentages for variables representing medical history

Variable	Categories	Numbers^a^	%
**Rehabilitation type**	Home	35	27.2
	Hospital	93	72.8
**Pattern of CHD**	PTCA	88	68.7
	CABG	20	15.65
	Angina	20	15.65
**CHD incidence time**	< 5 years	74	57.7
	5–10 years	37	28.5
	11–20 years	14	11.4
	> 20 years	3	2.4
**Disorders**	Respiratory problems	7	5.7
	Hypertension	42	33.0
	Diabetes mellitus	34	26.4
	Peripheral vascular disease	28	21.7
	Renal problems	4	2.8
	Hyperlipidaemia	12	9.4
	Others	1	1
**Emergency admission or hospitalization due to CHD**	Yes	101	78.9
	No	27	21.1

*CHD* coronary heart disease; *PTCA* percutaneous transluminal coronary angioplasty; *CABG* coronary artery bypass graft

^a^There were missing data on some of the variables

### CAM usage among study participants

A total of 59 (45.9%) participants used CAM for their heart problems; 125 (97.8%) patients used CAM because it had fewer side effects. Tables 3 and 4 show CAM methods used by patients with CHD, along with the frequencies and percentages of usage. Multiple vitamins and minerals (in a range of 2–15.4%) were among CAM methods used. The most common vitamins and minerals used were vitamin B complex (*n* = 20, 15.4%), cobalamin (*n* = 18, 13.7%), vitamin D (*n* = 17, 13.5%), iron (*n* = 17, 13.5%), and vitamin C (*n* = 15, 11.8%). More around 50% of the respondents used thyme, chamomile and medical herbs, and these were the highest herbs used (Table 3). Essential oils and Hijama were used by more than 20% of the respondents, and they were the most commonly used types in the body and traditional alternative medicine category, while religious practices were the most commonly used of all CAM methods (Table 4).

**Table 3:** Frequencies of usage for vitamins, minerals, diet and herbs

Type of CAM	Never	Twice at most	3–6 times	> 6 times	Cannot remember
**Vitamins and minerals**					
Vitamin E	90.4%	7.7%	1.9%	0.0%	0.0%
Folate	96.1%	2.0%	2.0%	0.0%	0.0%
Magnesium with zinc	98.0%	2.0%	0.0%	0.0%	0.0%
Omega-3 fatty acids	90.2%	2.0%	2.0%	5.9%	0.0%
Niacin	94.0%	2.0%	2.0%	2.0%	0.0%
Iron	86.5%	3.8%	3.8%	5.8%	0.0%
Vitamin C	88.2%	7.8%	2.0%	2.0%	0.0%
Calcium phosphate	94.0%	4.0%	0.0%	2.0%	0.0%
Cobalamin	86.3%	9.8%	2.0%	2.0%	0.0%
Vitamin D	86.5%	5.8%	5.8%	1.9%	0.0%
Vitamin B6	94.0%	4.0%	0.0%	2.0%	0.0%
Vitamin A	94.2%	3.8%	1.9%	0.0%	0.0%
Vitamin B complex	84.6%	7.7%	3.8%	3.8%	0.0%
**Diet and herbs**					
Medical herbs	43.4%	28.3%	11.3%	17.0%	0.0%
Honey	46.3%	25.9%	11.1%	13.0%	3.7%
Onion	66.0%	15.1%	9.4%	9.4%	0.0%
Black seed	62.3%	15.1%	7.5%	15.1%	0.0%
Fenugreek	73.1%	21.2%	0.0%	5.8%	0.0%
Garlic	64.2%	15.1%	9.4%	11.3%	0.0%
Chamomile	51.9%	27.8%	11.1%	9.3%	0.0%
Liquorice root	90.0%	8.0%	2.0%	0.0%	0.0%
Holy basil	80.4%	11.8%	7.8%	0.0%	0.0%
Milk thistle	69.2%	11.5%	11.5%	5.8%	1.9%
Thyme	43.4%	24.5%	18.9%	13.2%	0.0%
Ginger	60.4%	20.8%	11.3%	7.5%	0.0%
Cinnamon	58.8%	15.7%	13.7%	9.8%	2.0%
Balsam pear	55.8%	15.4%	9.6%	15.4%	3.8%
Mushrooms	52.9%	23.5%	11.8%	11.8%	0.0%
Prickly pear	56.6%	13.2%	17.0%	11.3%	1.9%

*CAM* complementary and alternative medicine

**Table 4:** Frequencies of usage for body, traditional and religious practices

Body and traditional alternative medicine					
Type of CAM	Never	Twice at most	3–6 times	> 6 times	Cannot remember
Chinese or Oriental medicine	98.1%	1.9%	0.0%	0.0%	0.0%
Acupuncture	100.0%	0.0%	0.0%	0.0%	0.0%
Reflexology	100.0%	0.0%	0.0%	0.0%	0.0%
Aromatherapy	96.2%	1.9%	1.9%	0.0%	0.0%
Relaxation therapy	92.2%	3.9%	3.9%	0.0%	0.0%
Essential oils	77.4%	7.5%	11.3%	3.8%	0.0%
Massage	86.8%	5.7%	1.9%	5.7%	0.0%
Yoga	98.1%	0.0%	1.9%	0.0%	0.0%
Exercise*	83.0%	9.4%	5.7%	1.9%	0.0%
Ayurveda	100.0%	0.0%	0.0%	0.0%	0.0%
Chiropractic and osteopathic medicine	90.6%	5.7%	1.9%	1.9%	0.0%
Hijama	79.2%	15.1%	5.7%	0.0%	0.0%
**Religious practices**					
Supplication	12.0%	10.0%	26.0%	40.0%	12.0%
Prayer	20.0%	8.0%	20.0%	32.0%	20.0%
Reading holy books	14.0%	8.0%	24.0%	44.0%	10.0%
Zamzam water	30.0%	44.0%	20.0%	4.0%	2.0%
Exorcism	91.7%	6.3%	0.0%	0.0%	2.1%
Roquia	82.0%	8.0%	4.0%	4.0%	2.0%

*CAM* complementary and alternative medicine

### Socio-demographic and clinical factors associated with CAM usage

It was noticed that the place of residency and pattern of CHD were significantly associated with CAM usage (*p* = 0.039 and 0.044, respectively). Patients who lived in cities were more likely to use CAM as compared to those who lived in villages and camps. Furthermore, patients who underwent CABG were also more likely to use CAM as compared to those with angina as the only presenting symptom (Table 5).

**Table 5:** Distribution of studied population according to socio-demographic and clinical data that characterizes users versus non-users of CAM

Variable	Categories	Users ^a^ ***N*** = 59	Non-users^a^ ***N*** = 69	***P***-value
**Gender**	Male	43	43	0.193
	Female	16	26	
**Place of residency**	Village	25	43	0.039
	City	27	17	
	Refugee camp	7	9	
**Social status**	Married	56	60	0.224
	Single	0	3	
	Widowed	3	6	
**Age**	< 35	0	2	0.750
	36–50	10	9	
	50–65	30	34	
	> 65	22	21	
**Educational level**	High school	35	50	0.231
	Diploma	12	7	
	Bachelor’s degree	11	12	
	Higher studies	1	0	
**Income**	Less than 2000 NIS	28	39	0.405
	2000–5000 NIS	28	21	
	5000–10,000 NIS	5	3	
	More than 10,000 NIS	1	3	
**Health insurance**	No health insurance	2	3	0.972
	Governmental insurance	49	60	
	Private insurance	7	7	
**Smoking status**	Smoker	25	30	0.758
	Non-smoker	34	39	
**Alcoholic drinks**	Yes	1	4	0.627
	No	57	66	
**Emergency**	Yes	49	52	0.106
	No	8	19	
**Pattern of CHD**	PTCA	44	44	0.044
	CABG	12	8	
	Angina	4	16	
**Rehabilitation type**	Home	13	22	0.289
	Hospital	47	46	
**Period of CHD**	Less than 5 yrs.	34	40	0.457
	5–10 yrs.	14	23	
	11–20 yrs.	9	5	
	More than 20 yrs.	2	1	
**Obesity**	Underweight	1	0	0.311
	Normal	11	20	
	Overweight	28	26	
	Obese	22	20	

*CHD* coronary heart disease; *PTCA* percutaneous transluminal coronary angioplasty; *CABG* coronary artery bypass graft; *CAM* complementary and alternative medicine; *NIS* new Israeli shekel (1 New Israeli Shekel = 0.29 US Dollar)

^a^There were missing data on some of the variables

### Attitudes of patients with CHD towards CAM usage

The Siahpush score [14, 59] was used to assess patient’s attitudes towards CAM usage (Table 6).

**Table 6:** Attitudes toward CAM in patients with CHD

	Median	Q1	Q3	IQR
**Attitudes towards alternative medicine (max score = 25)**	14.69	13.00	16.00	3
**Dissatisfaction with medical outcomes (max score = 30)**	17.00	16.00	19.00	3
**Dissatisfaction with medical encounters (max score = 35)**	22.00	20.00	23.00	3
**Individual responsibility (max score = 15)**	15.00	12.00	15.00	3
**Natural remedies (max score = 30)**	23.00	21.00	25.00	4
**Holism (max score = 20)**	16.00	14.00	17.00	3
**Rejection of authority (max score = 20)**	15.00	14.00	17.00	3
**Consumerism (max score = 5)**	3.00	2.00	4.00	2

*CHD* coronary heart disease; *CAM* complementary and alternative medicine Q1: percentile25, Q3: percentile75, *IQR* Q3-Q1

On the other hand, no significant associations were found between age categories, patients’ gender, and their attitude toward CAM (Table 7).

**Table 7:** Attitudes of patients with CHD toward CAM by age and gender

Age category^a^		Attitudes towards alternative medicine	Dissatisfaction with medical outcomes	Dissatisfaction with medical encounters	Individual responsibility	Natural remedies	Holism	Rejection of authority	Consumerism
**< 50** ***N*** **= 19**	**Median**	14.00	17.00	21.83	15.00	23.18	16.00	15.00	3.00
	**Q1**	13.00	16.00	20.00	12.00	22.00	14.00	14.00	2.00
	**Q3**	16.00	19.00	22.00	15.00	25.00	17.00	17.00	4.00
**50–65** ***N*** **= 56**	**Median**	14.00	17.00	22.00	13.74	23.00	16.00	15.00	3.12
	**Q1**	13.00	15.00	20.00	12.00	22.00	14.50	14.00	2.00
	**Q3**	16.00	18.50	24.00	15.00	25.00	17.00	17.00	4.00
**> 65** ***N*** **= 38**	**Median**	15.00	16.50	21.92	15.00	22.00	17.00	16.00	3.00
	**Q1**	14.00	15.00	20.00	13.00	21.00	14.00	14.00	2.00
	**Q3**	16.00	19.00	23.00	15.00	25.00	17.00	17.00	4.00
***P*-value**^**b**^		0.711	0.593	0.348	0.257	0.917	0.805	0.852	0.503
**Gender**									
**Male** ***N*** **= 86**	**Median**	14.69	17.00	22.00	15.00	23.00	16.00	15.00	3.00
	**Q1**	13.00	15.00	20.00	12.00	22.00	14.00	14.00	2.00
	**Q3**	18.00	22.00	27.00	15.00	29.00	20.00	17.00	5.00
**Female** ***N*** **= 42**	**Median**	14.35	17.00	22.00	15.00	23.00	16.00	15.17	3.00
	**Q1**	13.00	16.00	20.00	12.00	21.00	14.00	14.00	2.00
	**Q3**	17.00	22.00	25.00	15.00	28.00	19.00	17.00	5.00
***P*-value**^**c**^		0.569	0.653	0.625	0.668	0.898	0.876	0.717	0.827

*CAM* complementary and alternative medicine; *N* Number; *Q1* percentile25, *Q3* percentile75

^a^There were missing data on some of the variables

^b^*Kruskal–Wallis test*

^c^*Mann-Whitney U test*

In addition, Table 8 shows the association between the patient’s attitude toward CAM and CAM usage. The table shows a significant association between CAM usage and attitudes toward both alternative medicine and natural remedies (*p* = 0.011 and 0.044, respectively), which indicates that people with positive attitudes toward alternative medicine and natural remedies were found to be more likely to use CAM.

**Table 8:** Association between CAM usage and attitudes toward CAM

	CAM users ***N*** = 59 Median [Q1-Q3]	CAM non-users ***n*** = 69 Median [Q1-Q3]	***P***-value
Attitudes towards alternative medicine	14 [13–15]	15 [14–16]	0.011
Dissatisfaction with medical outcomes	17 [16–19]	17 [16–19]	0.883
Dissatisfaction with medical encounters	22 [20–23]	22 [20–24]	0.573
Individual responsibility	15 [12–15]	14 [12–15]	0.281
Natural remedies	24 [22–25]	22 [21–24]	0.044
Holism	16 [14–17]	16 [14–17]	0.979
Rejection of authority	15 [14–17]	15 [14–17]	0.985
Consumerism	3 [2–4]	3 [2–4]	0.114

*CAM* complementary and alternative medicine, *Q1* percentile25, *Q3* percentile75

### Key points regarding CAM usage

This section describes the points of view of respondents regarding CAM. Most of the respondents reported that they had the recommendation to use CAM from either a friend (*n* = 48, 37.5%) or a family member (*n* = 32, 25%). In all, 77 (55.8%) respondents said they got their CAM from an alternative health practitioner, 42.3% (*n* = 54) said they got it from a food store, and the rest (*n* = 3, 1.9%) said they got it from a pharmacy. The great majority (*n* = 117, 91.2%) of the respondents reported that they spent less than 200 New Israeli shekel (NIS) *(1 NIS = 0.29 US Dollars)* per month on CAM, while the rest said they spent more (*n* = 11, 8.8%). A great majority of patients (*n* = 120, 94.2%) reported that they did not experience any side effects when they used CAM. On the other hand, 76 (59.2%) of the respondents said they have a regular cardiologist visit to help them manage their CHD. In all, 107 (83.6%) patients who said they get help from a cardiologist said they have never discussed it with the cardiologist they get help from. When asked if they have visited a general practitioner or cardiologist in the last 3 months, 77 (60.2%) respondents reported that they visited them for heart problems, 11 (8.5%) reported that they visited them for reasons other than a heart problem. Furthermore, when asked about visiting any alternative health practitioner in the last 3 months, 22 (17.2%) reported that they have visited a practitioner for their heart problems and 3 (2.6%) reported that they did but for different reasons. On the other hand, 83 (64.9%) respondents said that they would consider using CAM in the future to help treat their CHD if they had positive information about its benefits from their healthcare provider, while 21 (16.2%) were not sure to use it. Table 9 shows the points of view regarding CAM usage.

**Table 9:** Points of view regarding CAM

Variable		Frequency	Percent
**Who recommended CAM?**	Doctor	7	5.4
	Friend	48	37.5
	Advertisement	18	14.3
	Naturopath	7	5.4
	Family member	32	25.0
	Internet	12	8.9
	Nurse	2	1.8
	Chiropractor	2	1.8
**Where do you get your CAM from?**	Health food store	54	42.3
	Pharmacy	3	1.9
	Alternative health practitioner	71	55.8
**How much money do you spend on CAM per month?**	< 200 NIS	117	91.2
	200–500 NIS	11	8.8
**Have you had any side effects from the use of CAM?**	Yes	3	1.9
	No	120	94.2
	don’t know	5	3.8
**Do you have a regular cardiologist who helps to manage your CHD?**	Yes	76	59.2
	No	52	40.8
**Have you discussed using CAM therapies with your cardiologist?**	Yes	21	16.4
	No	107	83.6
**Have you visited a general practitioner or cardiologist in the last 3 months?**	Yes, for my heart problems	77	60.2
	Yes, but not for my heart problems	11	8.5
	No	40	31.4
**Have you visited any alternative health practitioner in the last 3 months?**	Yes, for my heart problems	22	17.2
	Yes, but not for my heart problems	3	2.6
	No	103	80.2
**If you do not currently use CAM, would you consider using it to help treat your CHD in the future if you had positive information about its benefits from your healthcare provider?**	Yes	83	64.9
	No	24	18.9
	Not sure	21	16.2

*CHD* coronary heart disease; *CAM* complementary and alternative medicine; *NIS* new Israeli shekel (1 New Israeli Shekel = 0.29 US Dollar)

## Discussion

Currently, CVD is considered the leading cause of mortality and morbidity in Palestine as compared to other illnesses. In addition to being a worldwide burden on health systems, in 2010, CVD was responsible for 25% of deaths as reported by the Palestinian Ministry of Health [8]. A large portion of the Palestinian population uses CAM for a wide variety of health problems other than CHD, such as diabetes mellitus, hypertension, kidney problems, and asthma, and for the preservation of overall health, despite it not being largely supported by the medical community. In this study, it was found that 45.9% of patients with CHD have used CAM for their heart disease. In comparison to other studies abroad, for example, the prevalence of CAM usage among patients with CHD in Trinidad was 56.2% [13], the prevalence in the UK was 31.7% [14] and the prevalence in Texas was 54% [65], which indicates the proportionally high usage of CAM for the management of heart problems among Palestinians. A systematic review of current literature showed that the prevalence of CAM use ranged between 5 and 74.8% [66]. Another systematic review found that the prevalence of CAM use in cardiac patients ranged from 4 to 61% [63].

Regarding the reasons behind CAM usage, 125 (97.8%) respondents reported using it because it has fewer side effects as compared to old-fashioned drugs according to their experience, despite the lack of supporting evidence for its benefits. This can be attributed to the vast influences of traditions and social media on our society. Only 7 (5.4%) of the recommendations for CAM usage came from physicians. As reported in the results, different kinds of medical herbs, especially hawthorn, rosemary, and anise, have been widely used by the respondents, with 22 (17%) CAM users reporting using it more than six times a week, while black seed, cinnamon, honey, and thyme had a little over 10% usage among CAM users six times a week. Body and alternative medicine practices were the least popular CAM methods used among patients with CHD, which is most probably due to lack of awareness on the subtypes of this group. It is worth mentioning that Hijama and essential oils were the most used and well-known methods in the body and traditional alternative medicine category; with 26 (20%) respondents used them two times weekly, as opposed to religious practices, such as reading the holy book and supplication, with 51 (40%) respondents used them six times weekly. This was a highly popular method among CAM users, as they started to use religious practices as a spiritual therapy more frequently after the onset of their disease. This result was similar to that of a study conducted in Saudi Arabia where the most commonly employed practices were spiritually based methods, such as prayer and reading the Quran alone or on water, followed by herbs, honey, and dietary products [67].

The majority of the respondents in this study were found to be non-working males aged between 51 and 65 years, with most of them being urban dwellers of low socioeconomic status (less than 2000 NIS per month). However, regarding the socio-demographic variables, such as age and gender, obtained in this study, there was no significant association between CAM usage and patients with CHD, except for a place of residency (*p* = 0.039) and pattern of CHD (*p* = 0.044). It was noticed that city residents were more likely to use CAM for their CHD as compared to residents of villages and refugee camps. It’s also worth noting that patients who underwent PTCA and CABG had a higher chance of using CAM as compared to patients presenting with angina as the only presenting symptom, which had a negative relationship with CAM usage. Furthermore, in the UK study, they reported that patients post-revascularization had a positive association with CAM usage (with *p* = 0.01 indicating the significance of the relationship) [14].

This study had the privilege of being the first one carried out in Palestine to have analysed the attitudes of patients with CHD and how it influenced CAM usage, with consideration of age and gender variables. It was found that a significant association between CAM usage and attitudes towards alternative and complementary medicine is present (*p* = 0.011), which is in addition to the significant association between the attitudes toward natural remedies and CAM usage (*p* = 0.044). In contrast, a similar study carried out in the UK where the Siahpush score was also used to determine the relationship between attitudes and CAM usage, and it found a significant relationship between CAM usage and holism as well as individual responsibility (*p* = 0.01 and 0.009, respectively) [14].

When shedding light on the mechanism by which CAM users were pointed in the direction of using non-traditional methods, it was found that the vast majority of the patients were advised to use CAM by friends, followed by family members and a very low percentage were being advised by doctors and by nurses. However, 64.9% of the respondents reported their future willingness to use CAM for control of their CHD if it was recommended by their physician or if any published evidence-based articles were supporting the benefits of CAM usage for CHD. This would give them the necessary incentive they need for the use of CAM, while 16.2% of respondents were not sure if they would use CAM, despite receiving additional support for its benefits.

Because of the lack of evidence on the hazards and side effects of CAM usage, the majority of the respondents either reported being afraid of unknown side effects or not having any side effects, which leads us to conclude that having clearer guidelines on when and how to use CAM methods safely and effectively will significantly increase its integration into daily practice. Regarding regular follow-up of the respondents with their physicians, 76 (59.2%) of them reported committing to regular visits with their attending cardiologist (no more than 3 months apart) to help manage their heart problems. Unfortunately, the number of respondents who actually talked about or consulted their physician about CAM usage was only 16.4% (*n* = 21) of the total sample. When asked about the reason behind the lack of disclosure about this topic, most of the respondents attributed it to being afraid or thinking that it was not a crucial part of their therapeutic journey.

### Strengths and limitations

This study is the first study to be carried out in a Palestinian territory that is concerned with CAM usage among patients with the most common cause of mortality and morbidity in Palestine (i.e., CHD). In addition, this study is the first study to have used the Siahpush score to measure attitudes towards the healthcare system in Palestine. This subject is still a relatively uncharted area of research, especially in Palestine, and due to its importance, we had to tread carefully in order to ensure adequate coverage of the important aspects of this subject and to accurately display its relation to other important variables in healthcare. The main challenges we faced during our study revolved around three main limitations. The first limitation was centred on the cross-sectional design, which assumed that the cause-effect relationship with the determinants could not be assessed. The second limitation was the small sample size, which may have impacted the precision of several analyses making it difficult to generalize the findings to the general population. The missing in some important variables were the other limitation of this study.

## Conclusions

In Palestine, CAM is noticed to be popular among CHD patients, despite the lack of evidence-based research supporting its potential benefits and side effects, especially those who have undergone PTCA and CABG procedures and among urban dwellers. Furthermore, attitudes towards both alternative medicine and natural remedies were significantly associated with CAM usage. We recommend increasing focus and spreading awareness on CAM usage between healthcare workers, policymakers, and patients with CHD in order to provide a clear evidence-based practice that would quantify the benefits and hazards of CAM usage.

Several CAM methods were found to be popular among patients with CHD in Palestine, especially religious practices, such as reading the holy book and supplication, and herbal medicine with an obvious preference for the use of medical herbs, black seed, anise, chamomile, balsam pear and thyme. Vitamins and minerals were also used but to a lesser extent, with body and traditional alternative medicine being the least used group of all the CAM methods. Finally, we hope that more attention will be brought to this subject for the crucial part of its integration with other parts of the multidisciplinary health approach aiming to promote better health, prevent health problems and treat patients effectively.

Based on our findings we recommend: 1) Stressing the important role of CAM usage in the multidisciplinary approach towards health promotion and as an integral part of treatment through increasing awareness between healthcare workers and policymakers, with an emphasis on primary care physicians as they are considered the first line of contact with patients. 2) Raising patient’s awareness regarding the need for full disclosure of all the CAM methods they use with their attending physician to discuss the potential benefits and hazards of CAM usage and how to use it as effectively as possible. 3) Recognizing the active role of research in this field that is directed at providing full and clear guidelines on the right way to use CAM by following world-class evidence-based practices. 4) Adopting the holistic and patient-centred approaches in clinical settings, which will ensure the patient’s satisfaction and comfort when confronting their physician, plus dedicating a department in hospitals that are concerned with CAM. As a result, patients will have a place to learn and discuss this subject carefully before acting on their own accord.

## Supplementary information

**Additional file 1.** Study questionnaires. This is the final version of the English version that was used to obtain data which will help to examine the use of complementary and alternative medicine (CAM) among patients with coronary heart disease, the reasons and factors influencing their use, and the types of CAM used.

## Data Availability

The datasets used for the current study are available from the corresponding author upon request.

## Abbreviations

IRB: Institutional Review Board
CAM: Complementary and alternative medicine
SPSS: Statistical Package for the Social Sciences
BMI: Body mass index
SD: Standard deviation
CHD: Coronary heart disease
PTCA: Percutaneous transluminal coronary angioplasty
CABG: Coronary artery bypass graft
NIS: New Israeli shekel
CVD: Cardiovascular disease
AHA: American Heart Association
Q1: Percentile25
Q3: Percentile75
IQR: Q3-Q1

## References

[1] The Centers for Disease Control and Prevention (CDC). Heart Disease in the United States. 2017. https://www.cdc.gov/heartdisease/facts.htm (accessed Feb 17 2018).

[2] Roth GA, Johnson C, Abajobir A, Abd-Allah F, Abera SF, Abyu G, Ahmed M, Aksut B, Alam T, Alam K (2017). Global, regional, and National Burden of cardiovascular diseases for 10 causes, 1990 to 2015. J Am Coll Cardiol.

[3] Buttar HS, Li T, Ravi N (2005). Prevention of cardiovascular diseases: role of exercise, dietary interventions, obesity and smoking cessation. Exp Clin Cardiol.

[4] Winkleby MA, Jatulis DE, Frank E, Fortmann SP (1992). Socioeconomic status and health: how education, income, and occupation contribute to risk factors for cardiovascular disease. Am J Public Health.

[5] Mozaffarian D, Benjamin EJ, Go AS, Arnett DK, Blaha MJ, Cushman M, Das SR, de Ferranti S, Despres JP, Fullerton HJ (2016). Executive summary: heart disease and stroke statistics--2016 update: a report from the American Heart Association. Circulation.

[6] Hanson MA, Fareed MT, Argenio SL, Agunwamba AO, Hanson TR (2013). Coronary artery disease. Prim Care.

[7] American Heart Association. Coronary Artery Disease - Coronary Heart Disease. 2015. http://www.heart.org/HEARTORG/Conditions/More/MyHeartandStrokeNews/Coronary-Artery-Disease%2D%2D-Coronary-Heart-Disease_UCM_436416_Article.jsp#.WlETrohx3b3 (accessed May 21 2020).

[8] Abu-Rmeileh NM, Shoaibi A, O'Flaherty M, Capewell S, Husseini A (2012). Analysing falls in coronary heart disease mortality in the West Bank between 1998 and 2009. BMJ Open.

[9] Ministry of Health. Health Annual Report Palestine 2014. 2015. http://www.moh.ps/Content/Books/kD3bquHr7jbwK9f6VQJAsLDCuckgEDlCZUFa9ssb62m9Eim2le562D_ECDSNEboZRJwc6HyiggSMzKUPMeDJa2vkBNlAdZOGlvNuS9CHKJjGO.pdf (accessed Mar 11 2017).

[10] Braun LA, Tiralongo E, Wilkinson JM, Spitzer O, Bailey M, Poole S, Dooley M (2010). Perceptions, use and attitudes of pharmacy customers on complementary medicines and pharmacy practice. BMC Complement Altern Med.

[11] Wilkinson JM, Jelinek H (2009). Complementary medicine use among attendees at a rural health screening clinic. Complement Ther Clin Pract.

[12] Calcagni N, Gana K, Quintard B (2019). A systematic review of complementary and alternative medicine in oncology: psychological and physical effects of manipulative and body-based practices. PLoS One.

[13] Bahall M (2015). Complementary and alternative medicine usage among cardiac patients: a descriptive study. BMC Complement Altern Med.

[14] Greenfield S, Pattison H, Jolly K (2008). Use of complementary and alternative medicine and self-tests by coronary heart disease patients. BMC Complement Altern Med.

[15] Arthur HM, Patterson C, Stone JA (2006). The role of complementary and alternative therapies in cardiac rehabilitation: a systematic evaluation. Eur J Cardiovasc Prev Rehabil.

[16] Chen XW, Serag ES, Sneed KB, Liang J, Chew H, Pan SY, Zhou SF (2011). Clinical herbal interactions with conventional drugs: from molecules to maladies. Curr Med Chem.

[17] Chen XW, Sneed KB, Pan SY, Cao C, Kanwar JR, Chew H, Zhou SF (2012). Herb-drug interactions and mechanistic and clinical considerations. Curr Drug Metab.

[18] Hu Z, Yang X, Ho PC, Chan SY, Heng PW, Chan E, Duan W, Koh HL, Zhou S (2005). Herb-drug interactions: a literature review. Drugs.

[19] Izzo AA, Ernst E (2009). Interactions between herbal medicines and prescribed drugs: an updated systematic review. Drugs.

[20] Barraco D, Valencia D, Riba AL, Nareddy S, Draus CBSN, Schwartz SM (2005). Complementary and alternative medicine (CAM) use patterns and disclosure to physicians in acute coronary syndrome patients. Complement Ther Med.

[21] Ai AL, Bolling SF (2002). The use of complementary and alternative therapies among middle-aged and older cardiac patients. Am J Med Qual.

[22] Ai AL, Peterson C, Bolling SF (1997). Psychological recovery from coronary artery bypass graft surgery: the use of complementary therapies. J Altern Complem Med.

[23] Whitworth J, Burkhardt A, Oz MC (1998). Complementary therapy and cardiac surgery. J Cardiovasc Nursing.

[24] Ackman M, Campbeel JB, Buzak KA, Tsuyuki RT, Montague TJ, Teo KK. Use of non-prescription medications by patients with congestive heart failure. Ann Pharmacother. 1999;33. 10.1345/aph.18283 10410177

[25] Liu EH, Turner LM, Liu SX, Klaus L, Choi LY, Whitworth J, Ting W, Oz MC (2000). Use of alternative medicine by patients undergoing cardiac surgery. J Thorac Cardiov Sur.

[26] Chagan L, Bernstein D, Cheng JWM, Kirschenbaum HL, Rozenfeld V, Caliendo GC, Meyer J, Mehl B (2005). Use of biological based therapy in patients with cardiovascular diseases in a university-hospital in New York City. BMC Complement Altern Med.

[27] Zick SM, Blume A, Aaronson KD (2005). The prevalence and pattern of complementary and alternative supplement use in individuals with chronic heart failure. J Card Fail.

[28] Decker C, Huddleston J, Kosiborod M, Buchanana DM, Stoner C, Jones A, Banerjee S, Spertus JA (2007). Self-reported use of complementary and alternative medicine in patients with previous acute coronary syndrome. In: Am J Cardiol. edn.

[29] Yeh GY, Davis RB, Phillips RS (2006). Use of complementary therapies in patients with cardiovascular disease. Am J Cardiol.

[30] Chu FY, Yan X, Zhang Z, Xiong XJ, Wang J, Liu HX (2013). Features of complementary and alternative medicine use by patients with coronary artery disease in Beijing: a cross-sectional study. BMC Complement Altern Med.

[31] Pharand C, Ackman ML, Jackevicius CA, Paradiso-Hardy FL, Pearson GJ (2003). Use of OTC and herbal products in patients with cardiovascular disease. Ann Pharmacother.

[32] Wood MJ, Stewart RL, Merry H, Johnstone DE, Cox JL (2003). Use of complementary and alternative medical therapies in patients with cardiovascular disease. Am Heart J.

[33] Leung YW, Tamim H, Stewart DE, Arthur HM, Grace SL (2007). The prevalence and correlates of mind-body therapy practices in patients with acute coronary syndrome. In: Complement Ther Med*.* edn..

[34] Martinez-Selles M, Garcia Robels JA, Munoz R, Serrano JA, Frades E, Dominguez Munoa M, Almendral J (2004). Pharmacological treatment in patients with heart failure: patients knowledge and occurrence of polypharmacy, alternative medicine and immunizations. Eur J Heart Fail.

[35] Kristoffersen AE, Sirois FM, Stub T, Hansen AH (2017). Prevalence and predictors of complementary and alternative medicine use among people with coronary heart disease or at risk for this in the sixth Tromso study: a comparative analysis using protection motivation theory. BMC Complement Altern Med.

[36] Than M, Anam A, Nurfarahi K, Asma A, Hayati M (2019). Knowledge, use of complementary alternative medicine and health-related quality of life among cardiovascular disease patients. Food Res.

[37] Huber R, Koch D, Beiser I, Zschocke I, Lueedtke R. Experience and attitudes towards CAM-a survey of internal and psychosomatic patients in a German university hospital. Altern Ther Health Med. 2004;10. 14727497

[38] Sawalha AF (2007). Complementary and alternative medicine (CAM) in Palestine: use and safety implications. J Altern Complement Med.

[39] Ali-Shtayeh MS, Jamous RM, Jamous RM, Salameh NM (2013). Complementary and alternative medicine (CAM) use among hypertensive patients in Palestine. Complement Ther Clin Pract.

[40] Ali-Shtayeh MS, Jamous RM, Jamous RM (2012). Complementary and alternative medicine use amongst Palestinian diabetic patients. Complement Ther Clin Pract.

[41] Zyoud SH, Al-Jabi SW, Sweileh WM, Tabeeb GH, Ayaseh NA, Sawafta MN, Khdeir RL, Mezyed DO, Daraghmeh DN, Awang R (2016). Use of complementary and alternative medicines in haemodialysis patients: a cross-sectional study from Palestine. BMC Complement Altern Med.

[42] Ben-Arye E, Hamadeh AM, Schiff E, Jamous RM, Dagash J, Jamous RM, Agbarya A, Bar-Sela G, Massalha E, Silbermann M (2015). Compared perspectives of Arab patients in Palestine and Israel on the role of complementary medicine in cancer care. J Pain Symptom Manag.

[43] Ali-Shtayeh MS, Jamous RM, Jamous RM (2011). Herbal preparation use by patients suffering from cancer in Palestine. Complement Ther Clin Pract.

[44] Jaradat NA, Al-Ramahi R, Zaid AN, Ayesh OI, Eid AM (2016). Ethnopharmacological survey of herbal remedies used for treatment of various types of cancer and their methods of preparations in the West Bank-Palestine. BMC Complement Altern Med.

[45] Sawalha AF, Sweileh WM, Zyoud SH, Jabi SW (2008). Self-therapy practices among university students in Palestine: focus on herbal remedies. Complement Ther Med.

[46] Zyoud SH, Abd-Alhafez AB, Hussein AO, Abu-Shehab IS, Al-Jabi SW, Sweileh WM (2014). Patterns of use of medications, herbal products and nutritional supplements and polypharmacy associating factors in Palestinian geriatric patients. Eur Geriatr Med.

[47] Ali-Shtayeh MS, Jamous RM, Jamous RM (2015). Plants used during pregnancy, childbirth, postpartum and infant healthcare in Palestine. Complement Ther Clin Pract.

[48] Al-Ramahi R, Jaradat N, Adawi D (2013). Use of herbal medicines during pregnancy in a group of Palestinian women. J Ethnopharmacol.

[49] Jazieh AR, Al Sudairy R, Abulkhair O, Alaskar A, Al Safi F, Sheblaq N, Young S, Issa M, Tamim H (2012). Use of complementary and alternative medicine by patients with cancer in Saudi Arabia. J Altern Complement Med.

[50] Akhu-Zaheya LM, Alkhasawneh EM (2012). Complementary alternative medicine use among a sample of Muslim Jordanian oncology patients. Complement Ther Clin Pract.

[51] Ching SM, Zakaria ZA, Paimin F, Jalalian M (2013). Complementary alternative medicine use among patients with type 2 diabetes mellitus in the primary care setting: a cross-sectional study in Malaysia. BMC Complement Altern Med.

[52] Saad B, Azaizeh H, Said O (2005). Tradition and perspectives of Arab herbal medicine: a review. Evid Based Complement Alternat Med.

[53] Azaizeh H, Saad B, Khalil K, Said O (2006). The state of the art of traditional Arab herbal medicine in the eastern region of the mediterranean: a review. Evid Based Complement Alternat Med.

[54] Ali-Shtayeh MS, Yaniv Z, Mahajna J (2000). Ethnobotanical survey in the Palestinian area: a classification of the healing potential of medicinal plants. J Ethnopharmacol.

[55] Ministry of Health, Palestinian health information center. Health Status, Palestine, 2018. 2019. http://site.moh.ps/Content/Books/fE4zsafxsjNVhJntidJnqnnEHUibMuC1NYu66TNEmoNUJ1ZxeRcCm3_Iei1j8d4YesYKxRyEhD6PZqdxzBa4z91pIhALGXoDGEhlEIPai9X9O.pdf (accessed 7 Jan 2020).

[56] Samara AM, Barabra ER, Quzaih HN, Zyoud SH (2019). Use and acceptance of complementary and alternative medicine among medical students: a cross sectional study from Palestine. BMC Complement Altern Med.

[57] Shraim NY, Shawahna R, Sorady MA, Aiesh BM, Alashqar GS, Jitan RI, Abu Hanieh WM, Hotari YB, Sweileh WM, Zyoud SH (2017). Community pharmacists' knowledge, practices and beliefs about complementary and alternative medicine in Palestine: a cross-sectional study. BMC Complement Altern Med.

[58] Siahpush M (1998). Postmodern values, dissatisfaction with conventional medicine and popularity of alternative therapies. J Sociol.

[59] Marinac JS, Buchinger CL, Godfrey LA, Wooten JM, Sun C, Willsie SK (2007). Herbal products and dietary supplements: a survey of use, attitudes, and knowledge among older adults. J Am Osteopath Assoc.

[60] Zyoud SH, Al-Jabi SW, Sweileh WM (2015). Scientific publications from Arab world in leading journals of integrative and complementary medicine: a bibliometric analysis. BMC Complement Altern Med.

[61] Jang A, Kang DH, Kim DU (2017). Complementary and alternative medicine use and its association with emotional status and quality of life in patients with a solid tumor: a cross-sectional study. J Altern Complement Med.

[62] Pearson H, Fleming T, Chhoun P, Tuot S, Brody C, Yi S (2018). Prevalence of and factors associated with utilization of herbal medicines among outpatients in primary health centers in Cambodia. BMC Complement Altern Med.

[63] Grant SJ, Bin YS, Kiat H, Chang DH (2012). The use of complementary and alternative medicine by people with cardiovascular disease: a systematic review. BMC Public Health.

[64] Siahpush M (1999). Why do people favour alternative medicine?. Aust N Z J Public Health.

[65] Krasuski RA, Michaelis K, Eckart RE (2006). The cardiovascular patient's perceptions of complementary and alternative medicine. Clin Cardiol.

[66] Frass M, Strassl RP, Friehs H, Mullner M, Kundi M, Kaye AD (2012). Use and acceptance of complementary and alternative medicine among the general population and medical personnel: a systematic review. Ochsner J.

[67] Alrowais NA, Alyousefi NA (2017). The prevalence extent of complementary and alternative medicine (CAM) use among Saudis. Saudi Pharm J.

