# Supplementary material for: Complementary and alternative medicine use in coronary heart disease patients: a cross-sectional study from Palestine
Source: BMC Complement Med Ther. 2020 Jul 20;20:231. doi: 10.1186/s12906-020-03028-w (PMC7372840; doi:10.1186/s12906-020-03028-w)
Supplement: Supplementary file 1 — Additional file 1. Study questionnaires. This is the final version of the English version that was used to obtain data which will help to examine the use of complementary and alternative medicine (CAM) among patients with coronary heart disease, the reasons and factors influencing their use, and the types of CAM used. [file 12906_2020_3028_MOESM1_ESM.doc]

**Additional file 1: Study questionnaires. This is the final version of the English version that was used to obtain data which will help to examine the use of complementary and alternative medicine (CAM) among patients with coronary heart disease, the reasons and factors influencing their use, and the types of CAM used.**

| **Part one: Sociodemographic variables** |
| --- |

| **Age** | ………………..Year | | | | | | | | | | |
| --- | --- | --- | --- | --- | --- | --- | --- | --- | --- | --- | --- |
| **Gender** | 1. Male | | 2. Female | | | | | | | | |
| **Height** | …….. kg | | | | | | | | | | |
| **Weight** | …….. m | | | | | | | | | | |
| **BMI** | ………. Kg / m^2^ | | | | | | | | | | |
| **Residency** | 1.City | 2.Village | | | | 3.Palestinian refugee camp | | | | | |
| **Education level** | 1.Elementary | | | | 2.Middle school | | 3.High school | | | | 4.Diploma |
|  | 5. Bachelor’s degree | | | | 6. Higher studies | | | | | | |
| **Work status** | 1.Not working | | | 2.Governmental employee | | | | | 3.Private sector employee | | |
|  | 4. Retired | | | | | | | | | | |
| **Monthly income** | 1. Less than 2000 NIS | | 2. 2000-5000 NIS | | | 3. 5000-10000 NIS | | | | 4. More than 10000 NIS | |
| **Birth place:** | 1.Palestine | 2.Abroad | | | | | | | | | |
| **Health insurance** | 1. No insurance | | 2. Governmental insurance | | | | | 3. Private insurance | | | |

| **Part two: Medical history** |
| --- |

| **Rehabilitation type** | - Home | - Hospital | |
| --- | --- | --- | --- |
| **Pattern of CHD** | - PTCA | - CABG | - Angina |
| **CHD incidence time** | ……………….year | | |
| **Emergency admission or hospitalization due to CHD** | - No | - Yes | |
| **Do you have any chronic diseases?** | - No | - Cardiovascular disease - Thyroid disease - Hypertension - Diabetes Mellitus - Renal failure - Epilepsy - Asthma - Thalassemia - Other (mention): …………. | |
| **Do you smoke?** | - No | - Yes | |
| **Do you drink alcohol?** | - No | - Yes | |

| **Part three: CAM** |
| --- |

| **Vitamins and minerals** | | | | | |
| --- | --- | --- | --- | --- | --- |
| **Type of CAM** | **Never** | **Twice at most** | **3–6 times** | **> 6 times** | **Cannot remember** |
| Vitamin E |  |  |  |  |  |
| Folate |  |  |  |  |  |
| Magnesium with zinc |  |  |  |  |  |
| Omega-3 fatty acids |  |  |  |  |  |
| Niacin |  |  |  |  |  |
| Iron |  |  |  |  |  |
| Vitamin C |  |  |  |  |  |
| Calcium phosphate |  |  |  |  |  |
| Cobalamin |  |  |  |  |  |
| Vitamin D |  |  |  |  |  |
| Vitamin B6 |  |  |  |  |  |
| Vitamin A |  |  |  |  |  |
| Vitamin B complex |  |  |  |  |  |

| **Diet and herbs** | | | | | |
| --- | --- | --- | --- | --- | --- |
| **Type of CAM** | **Never** | **Twice at most** | **3–6 times** | **> 6 times** | **Cannot remember** |
| Medical herbs |  |  |  |  |  |
| Honey |  |  |  |  |  |
| Onion |  |  |  |  |  |
| Black seed |  |  |  |  |  |
| Fenugreek |  |  |  |  |  |
| Garlic |  |  |  |  |  |
| Chamomile |  |  |  |  |  |
| Liquorice root |  |  |  |  |  |
| Holy basil |  |  |  |  |  |
| Milk thistle |  |  |  |  |  |
| Thyme |  |  |  |  |  |
| Ginger |  |  |  |  |  |
| Cinnamon |  |  |  |  |  |
| Balsam pear |  |  |  |  |  |
| Mushrooms |  |  |  |  |  |
| Prickly pear |  |  |  |  |  |
| Fish oil |  |  |  |  |  |
| Probiotic |  |  |  |  |  |
| Carnitine |  |  |  |  |  |
| Soy bean |  |  |  |  |  |
| Sage |  |  |  |  |  |
| Peppermint |  |  |  |  |  |
| Anise |  |  |  |  |  |
| Cumin |  |  |  |  |  |
| Turmeric |  |  |  |  |  |
| Parsley |  |  |  |  |  |
| Green tea |  |  |  |  |  |
| Saffron |  |  |  |  |  |
| Rosemary |  |  |  |  |  |
| Ginseng |  |  |  |  |  |
| Olive oil |  |  |  |  |  |
| Castor oil |  |  |  |  |  |
| Grapeseed oil |  |  |  |  |  |
| Almond oil |  |  |  |  |  |
| Alovera |  |  |  |  |  |
| Cranberry |  |  |  |  |  |
| Others |  |  |  |  |  |

| **Body and traditional alternative medicine** | | | | | |
| --- | --- | --- | --- | --- | --- |
| **Type of CAM** | **Never** | **Twice at most** | **3–6 times** | **> 6 times** | **Cannot remember** |
| Chinese or Oriental medicine |  |  |  |  |  |
| Acupuncture |  |  |  |  |  |
| Reflexology |  |  |  |  |  |
| Aromatherapy |  |  |  |  |  |
| Relaxation therapy |  |  |  |  |  |
| Essential oils |  |  |  |  |  |
| Massage |  |  |  |  |  |
| Yoga |  |  |  |  |  |
| Exercise |  |  |  |  |  |
| Ayurveda |  |  |  |  |  |
| Chiropractic and osteopathic medicine |  |  |  |  |  |
| Hijama |  |  |  |  |  |
| Meditation |  |  |  |  |  |
| Dancing |  |  |  |  |  |
| Music |  |  |  |  |  |
| Hypnotherapy |  |  |  |  |  |
| Cupping |  |  |  |  |  |
| Detox |  |  |  |  |  |
| Spine manipulation |  |  |  |  |  |
| Others |  |  |  |  |  |

| **Religious practices** | | | | | |
| --- | --- | --- | --- | --- | --- |
| Supplication |  |  |  |  |  |
| Prayer |  |  |  |  |  |
| Reading holy books |  |  |  |  |  |
| Zamzam water |  |  |  |  |  |
| Exorcism |  |  |  |  |  |
| Roquia |  |  |  |  |  |
| Religious songs |  |  |  |  |  |

| **Part four: Attitudes toward CAM in patients with CHD*** |
| --- |

|  |  |  |  |  |
| --- | --- | --- | --- | --- |
| Attitudes towards alternative medicine (max score = 25) |  |  |  |  |
| Dissatisfaction with medical outcomes (max score = 30) |  |  |  |  |
| Dissatisfaction with medial encounters (max score = 35) |  |  |  |  |
| Individual responsibility (max score = 15 ) |  |  |  |  |
| Natural remedies (max score = 30) |  |  |  |  |
| Holism (max score = 20) |  |  |  |  |
| Rejection of authority (max score = 20) |  |  |  |  |
| Consumerism (max score = 5) |  |  |  |  |

*These scales were designed to assess patient’s attitudes towards the use of CAM based on the Siahpush score. Permission to use the tool was sought and granted from Siahpush, 1998. (Siahpush, M. (1998). Postmodern values, dissatisfaction with conventional medicine and popularity of alternative therapies. *Journal of Sociology*, *34*(1), 58–70.)

| **Part five: Points of view regarding CAM** |
| --- |

| Variable | |
| --- | --- |
| Who recommended CAM? | - Doctor - Friend ‎ - Advertisement - Naturopath - Family member - Internet - Nurse - Chiropractor‎ |
| Where do you get your CAM from? | - Health food store - Pharmacy - Alternative health practitioner |
| How much money do you spend on CAM per month? | - …………… NIS |
| Have you had any side effects from the use of CAM? | - Yes - No - don't know |
| Do you have a regular cardiologist who helps to manage your CHD? | - Yes - No |
| Have you discussed using CAM therapies with your cardiologist? | - Yes - No |
| Have you visited a general practitioner or cardiologist in the last 3 months? | - Yes, for heart problems - Yes, but not for heart problems - No |
| Have you visited any alternative health practitioner in the last 3 months? | - Yes, for heart problems - Yes, but not for heart problems - No |
| If you do not currently use CAM, would you consider using it to help treat your CHD in the future if you had positive information about its benefits from your healthcare provider? | - Yes - No - Not sure |
